# On the Moral Therapeutics of London

**Published:** 1859-04-01

**Authors:** 


### Art. VI.—ON THE MORAL THERAPEUTICS OF LONDON.

Chaucer, in a single sentence, pricked the core of all therapeutical science. In his Knight's Tale, he tells us, when the leech's efforts had proved unavailing for the relief of the dying Arcite, that—

> "Certainly ther nature wol not werche,
> Farewel physike."

This, which is true of the therapeutics of the body, is also true of the therapeutics of the soul. Our ability to aid in each case is governed by the power we possess of rousing into action the laws which regulate our being if dormant, or restraining, controlling, or guiding them if errant. From this it would follow that a system of moral therapeutics will be effective in proportion as it is based upon a correct appreciation and knowledge of the laws which dominate our moral life. To examine these laws and trace them to their ultimate consequences is a function of the psychologist; and hence it happens that psychology should become the right-hand helper of morality, supporting it when drooping, aiding it when stumbling, recalling it when wandering, and lighting with a brighter light the rugged pathway along which it had long successfully struggled, guided by experience alone, when psychology and kindred sciences were still floundering in the mud. Has psychology probed so deeply the springs of our moral life, that henceforth it may aid in guiding us more surely along the strait paths of virtue? We believe that it has. It teaches us (if we read its lessons aright) that the foundation of our moral life rests in the depths of our intuitional consciousness; that man's soul, by virtue of its inherent properties, on contact with human life, spontaneously becomes conscious of the moral quality of human actions; that this primary, direct, and immediate knowledge supplies the material upon which the reason works, and evolves those great antithetical ideas which form the basis of all systems of morality—right and wrong, good and evil, virtue and vice. Further, it teaches us that the primary, intuitive knowledge of good and evil is common to all men, is generic; that the subsequent evolution by aid of the reason is individual: that the one, the fundamental consciousness of right and wrong, is essentially the same in all individuals, among all races, and under all climes—is universal; that the other, the logical development and construction of the primary notions, partakes of the character of the individual, and varies with this character.

The intuitive action of the soul underlies all intellectual action, supplying not only the groundwork of moral truth, but of all truth of a higher kind: this is indeed the focus, the vital centre of all intellectual action, and it matters not how wide the reason may have flown, how eccentric its flights may have been, sooner or later it has acknowledged the centre around which it revolves.

This doctrine rests the basis of moral truth upon self-experience, and it is consistent with experience. Moreover, it is consistent also with our physiological knowledge; for in this intuitive action of the soul, what have we more than a manifestation of that general law which is witnessed in action throughout the whole of the organic kingdom, and beyond an expression of which our knowledge has not enabled us to pass—that the results of the specific action of an organism are due to the harmonious relation which exists between that organism and the influences which call its peculiar properties into operation? We know the phenomena by which these results are made manifest; we can trace a speciality of function to a speciality of organisation, and may surmise that the modifications of the one are correlative with modifications of the other; but we cannot get further than the fact that there exists, potentially, in the organism that capacity of action which is manifested upon exposure to certain influences. And thus of the organism through which the conscious soul of man is manifested, we say that in it rest, potentially, those modes of action which become apparent on its impact with the outer life, and to the primary, direct, and spontaneous phenomena of which, in relation to the higher manifestations of intelligence, the term *intuitive* has been applied.

In the ordinary phenomena of intelligence the intuitive and reasoning portions of our knowledge are invariably blended together, but the one has no necessary relation of development to the other. The intuitive perception of moral truth may be as complete and clear in the uncultured as the cultured mind; and, on the other hand, the logical powers may be developed to their highest degree, while the moral intuitions may be obscure and grovelling. No limit can be placed to the variable relationship of the one to the other which may be found in different individuals; but whether we seek to ascertain the sources of these variations by observations alone, or by deduction from the known laws of man's intellectual development, we learn that both the intuitive and reasoning faculties are inextricably linked with our physical being, and are liable to be obscured, or warped, or even extinguished, by changes going on within it. In truth, both the development of the one faculty and the other, and the causes of the modifications they undergo, are questions which come largely within the domain of pure physiology, and by this science we are taught that the degree of development is determined in a great measure by the physical and intellectual media in which we are placed; that the mind *grows* to the modes of action induced in it; that the inordinate development of any one faculty is accompanied by a corresponding degeneration of other faculties; that the aptitude to perception may be modified and obscured by transitory changes going on both within and without the body; that, in short, the mind reflects mainly the culture to which it has been submitted, except, as too often happens, it has been scarred by some hereditary flaw. The mind, indeed, is to the body what the flower is to the plant; and as by the careful and thoughtful tending of the gardener the petals of a corolla may be multiplied in number, increased in amplitude, and become tinted with richer and brighter colours, so may the mind, by proper nurture, become healthier in its intuitive capacity, and more gloriously habited in its thoughts: but in the one case, as the other, this is not to be effected in solitary individuals or in a single period; it is to be brought about through successive generations. In man this capacity of progressive intellectual development is the characteristic of his intuitional, and not of his reasoning faculty; for, as Morell has written—

"The laws and rules of formal logic are exactly the same *now* that they were in the time of Aristotle; and the application of these to any class of facts which may be known to each age, is made in every case in the same manner, and much about to the same degree. Here no *progress* is observable; the diversity of logical power in different ages is no greater than what may be found among individuals in the same era. But if we turn from the logical to the intuitional consciousness, here, instead of a fixed result, we find a perpetual motion, and regarding mankind *as a whole*, a constant progression. In one country, for instance, we find musical sensibility greatly in advance; in another, the perception of beauty through the eye (as was the case among the Greeks) has arrived at a high degree of perfection; in other instances, there is a peculiar awakening of the moral or of the religious consciousness: in a word, whenever we find our direct intentions coming into operation, *there* we find a kind of *vital development*, not confined to individual minds, but flowing generally through the consciousness of the mass. In this intuitional life, moreover, *progress* is as essential as in every other kind of vitality. Here stagnation indicates disease and decay; for so sure as man was created for an ultimate end—so sure as he was intended to arrive at even higher attainments in everything great and good, must his pathway be perpetually upwards, and the whole sensibilities of his nature come more and more into harmony with the Divine nature, with the *life of God*."—(*Philosophy of Religion*, 1849, p. 56.)

Here, then, we may again take up the notion with which we at first started—that a system of moral therapeutics, to be effective, must be based upon a knowledge of the laws of man's moral life; and if the views which we have advanced be correct, it would follow that the system should aim at rousing into freer action and developing more completely our moral intuitions—the fountain-head of our moral life; and that whatever aided towards that end should have a fitting place in the *materia medendi* of the soul.

Let us now endeavour to ascertain if these views have anything in common with, and if they throw any light upon, the practice of morality.

In a recent number of this Journal (*No. XII. New Series*) we attempted to show the principal circumstances which co-operated in the production of and fostered the abnormal conditions of morality—the vice—existing in of the metropolis. We traced the influence of filthy and crowded dwellings; the absence of the means of cleanliness and decency; the indiscriminate herding together of the young of both sexes; ignorance; and the thousand-and-one sensual temptations of a metropolitan life: we showed how, by association (slight and indirect though it might be) and contiguity, the vices of one class radiated among other classes of the population; how the immorality of St. Giles's and Clerkenwell often bore its fruit in the nurseries of St. James's and Bloomsbury, and the conventional morality of Bloomsbury and St. James's formed a *quasi* justification for the overt villany of St. Giles's and Clerkenwell: moreover, we pointed out that crime and every other form of manifest vice was the scum thrown to the surface of a low grade of morality which deeply infected every class of society, and the foundation of which was chiefly laid in childhood.

But while we drew broadly the dark picture of the vice of this great city—a picture which might well dishearten the most sanguine moralist—we left almost untouched those lights which might be found glimmering, be it ever so fitfully, in the profoundest sloughs of vice. However debauched the mind—however overlaid with villany, or darkened by ignorance and sensuality—unless disease have preyed upon the delicate organ through which it is manifested, we invariably find it swayed by the deep intuitions of moral truth. Distorted though these may be, still they exist and govern the individual; and from the innermost recesses to the outermost verge of vice, we find that it is ruled by its own codes of right and wrong, good and evil.

And in this consists the great hope of the moralist, for it is the flickering of the smothered fire which he has to animate and fan into a pure flame—a mighty task, and one at which the wisest and best will often blench!

If we glance over the therapeutical means at our command by which this task may be effected, we find, foremost in place, religion (for the religion of Christianity includes its morality, and the one may not be dissociated from the other), the restraints of the law, and the education of the intellect. These are the three great moral therapeutical agents; the first applying directly to, and its effectiveness depending upon, man's intuitive knowledge of good and evil; the second and third acting indirectly. Around these three agents there are grouped, in addition, numberless charities, all tending to ameliorate the social condition of the population, and to raise the moral standard.

The metropolis is chequered with churches and places of worship; an army of police haunts its streets, to maintain order and suppress crime; schools dot every alley and every thoroughfare; vast hospitals and dispensaries open their doors widely to the impoverished sick and maimed; not a form of misery or degradation exists, but hands are found stretched out to aid it: food is offered to the hungry, clothing to the naked, shelter to the homeless; baths and washhouses, built in the midst of the foulest districts, afford the means of and an inducement to cleanliness; model lodging-houses exist and are increasing in number, refuges from the horrible courts and bye-ways which exist in our brick-and-mortar jungles: there are sanctuaries for the fallen, banks for the provident; asylums for the aged, the blind, the deaf-mute, and the lunatic: every religious congregation forms an independent source of benevolence, which is extended to the depraved, the sick, and the dying; while private charity moves at large in the, alas! illimitable field which spreads out for its exercise.

Here, then, it would seem that what psychology would lay down as the true method of moral therapeutics, the practice of morality has already attained; for we find in play at one and the same moment all those influences which could be required for removing morbid conditions of and more fully developing the moral faculties.

But we must not too hastily imagine that these agencies, although existing at the same period, are closely linked together in their operation. Almost every agency is isolated in its position, and its efforts are directed solely to its individual aim, the general results upon morality, from the combined effects of the different agencies, being indirect and commonly unforeseen. Indeed, the general moral benefits arising from them are another illustration of those operations of man in society which have been well termed instinctive—operations in which apparently disconnected and often seemingly incoherent measures are found all tending to the production of certain general results not previously contemplated.

While, then, the highest teachings of psychological theory would point to the same practical results which have already been attained, however disconnectedly, by experience, it remains to be seen whether these results have been as effective in their application as theory would lead us to anticipate.

The data required for the complete answer to this important query, one which could only be dealt with thoroughly by a professed statist, we have not at our command. We have, however, a sufficiency of materials not only to show that the moral agencies we possess, rightly applied, are sufficient to achieve the object to which they are directed, but also to indicate the sources of their falling short of the point we desire. We might describe the vast moral advancement which has taken place within the metropolis during the last fifty years, and illustrate the effects produced by several of the most important moral agencies at different epochs, but we shall confine ourselves to a brief summary of a few of the results obtained by the late Mr. Fletcher in his elaborate examination of the moral statistics of the kingdom.—(*Journal of the Statistical Society*, vols. x., xi., xii.)

According to Mr. Fletcher, it would appear that the moral position of the two metropolitan counties (the moral character of which is governed almost entirely by the metropolis), in so far as this could be deduced from the statistical records accessible for 1839-44, was as follows :—

They possessed the greatest amount of education of any portion of the kingdom, and consequently the least amount of ignorance, this being 58.1 per cent. below the average of the kingdom ; they showed the least amount of improvident marriages (62·5 per cent. below the average), the least amount of bastardy (48·5 below the average), an amount of pauperism which was 12·5 per cent. below the average, the highest amount of savings in banks (55·6 above the average), but an amount of criminal commitments which was 11·3 per cent. above the average. The proportion of bastardy must be cast aside altogether, as, from the facilities which exist in the metropolis for concealing the illegitimacy of births, the number of bastards entered in the registers of births are much below the actual number existing. The number of criminal commitments (the return which above all others most persons are inclined to regard as the safest statistical criterion of the moral state of a community), notwithstanding that it is above the average, shows, when fully examined, in the strongest light the influence of the moral agencies now at work in the advancement of the moral and social condition of the metropolis.

"One of the most important results of Mr. Fletcher's inquiries was to show that in the thirty years 1811-41, the number of annual commitments had trebled, while the population had scarcely more than doubled; the increase of crime being thus six times faster than that of population. This is to be accounted for by the fact that the increase in criminality which occurred during the several great social disturbances affecting labour in the period referred to was propagated to a greater or less extent in the intervals of disturbance, indicating that the moral deterioration which is so apt to take place in periods of great social suffering is not immediately recovered from when the cause of suffering is withdrawn, but infects, more or less, subsequent periods. In Middlesex, however, during 1811-1841, the increase of commitments was only *one-sixth* of the general increase, or 63·4 per cent. instead of 319·5. The influence of the metropolis was, moreover, felt in the neighbouring counties: the increase in Surrey being only 189·5; Kent, 253·9; Herts, 261·5; and Essex, 309·0: while the increase in Sussex was 489·1; Buckingham, 534·1; and Bedford, 669·0. Influences, therefore, antagonistic to the increase of crime must have been at work in the metropolis and its neighbourhood.

"But, again, the criminality of a large town may be divided into two portions—that which has its birthplace in it, and that which *migrates* to it, being tempted there from the country by the greater field for its exertions. The crime born where it happens is chiefly that of a serious character (offences against the person, and malicious offences against property); the crime affected by migration consists mainly of offences against the person without violence, assaults, and miscellaneous offences. The first class of crime is that which most truly represents the actual tendency of a community to criminality, and it is shown that the "*excess of the more heinous and brutal* [*crimes*], *and those which are least affected by migration of the depraved, is always on the side of the greater ignorance.*" The metropolis, however, holds an exceptional position to the rest of the kingdom; for while between 1842-44 and 1845-47 there had been an actual diminution of serious crime throughout the kingdom to the extent of 17·3 per cent., in the metropolis there had been an actual increase of 6·4 per cent. Forgery and offences against the currency are, also, always in excess in the metropolis. These exceptions do not, however, disturb the remarkably slow progress of crime in the metropolis, and they are due to its being the select haunt of some of the most depraved characters."

The favourable position which the metropolis holds in its general social and criminal statistics, according to this brief survey, is to be attributed in a great measure to the degree of education existing within it, and to its excellent police; for the statistics not only of the metropolis, but also of the whole kingdom, make manifest how intimately the moral and social condition of a community is bound up with the degree of education prevailing in it, and the efficiency of its police. With the explicable exception of the metropolis mentioned, serious crime is universally in excess wherever ignorance is in excess; indeed, Mr. Neison goes further, and as the result of his examination of the statistics of crime in England, he concludes that—

"By adopting the test of education or instruction furnished by the marriage registers of the country, and . . . by so analysing the various districts and groups of counties that they differ in respect of education only, it is found that out of the twenty-two different combinations formed of the various districts in England and Wales, in every instance there is an excess of crime where there is the least education or instruction; and comparing the respective sections of each group of counties, it will be seen that there is an average excess of 25 per cent. of crime in the section of inferior education over that of higher education, and in some districts the excess is as much as 44 per cent.; that it is hence obvious that the very small amount of education, or rather instruction, implied by the test here adopted, has a powerful influence on the criminal calendar of the country."—(*Contributions to Vital Statistics*, 2nd ed., 1857, p. 405.)

Of the influence of the police, it may be said that while in the three years from 1842-44 to 1854-57 (the only period of steady decline in the number of commitments in the thirty years' criminal returns examined by Mr. Fletcher), the decline of the gross number of commitments was 18·6 per cent. in the policed counties, it was only 8·7 in the west of England and Wales, the decline in the whole kingdom being 13·2 per cent.

Notwithstanding that this general beneficial influence may be conceded to the agencies mentioned (for we need not dwell further upon the general statistics of the question: the *individual* benefits arising from education and the restraints of the law cannot for a moment be doubted), and although it may be admitted that the overt morality as well as the general moral fervour of the kingdom are of a higher grade now than was the case fifty years ago, it is hardly to be doubted that the legitimate effects of the moral agents of every class now in operation are not so manifest among the population at large, when the conventional morality (a great step in advance, by the way) which veneers the surface of society is picked off, as might be hoped for and reasonably expected; and it would seem that, notwithstanding the apparent soundness of our moral therapeutics both in principles and practice, we are rather holding at bay than actually overcoming the flood of vice with which we have to contend. Whence comes this? We believe that this doubtful position of morality is entirely due to the fact that the development of our moral agencies has not kept pace with the increase of population and the prodigiously growing wealth, and even mental cultivation, of the nation; that while, on every hand, the temptations to moral perversity have been increasing in consequence of spreading luxury, and greater facilities for indulgence, both physical and intellectual, that careful tuition of the moral faculties which should form the chief guard to perversion, and be the means of making increased wealth and a larger knowledge an increased benefit to our highest humanity, has not taken place to a like extent. The means of religious and moral tuition, and of education, have been wonderfully developed during the past fifty years; but has the development been in proportion to the requirements of the nation—has it not kept pace rather with the growing knowledge among the people of the absolute necessity of these means for the social and political welfare of the kingdom, and is not the development governed too much by notions of this kind than by notions of a higher and purer nature? Has not, indeed, a large portion of the comparatively recent great growth of religious and moral agencies been as much in luxury as in spirit? Not long ago, we listened to a homily addressed to a congregation of about five hundred souls, who were assembled in a splendid building that had cost 30,000*l.*, and which is a gem of architecture, although unfinished, and we were informed that 30,000*l.* more were required to complete it. Several priests officiated in the hour-and-a-half's service, a well-trained choir sang efficiently, and the prayers were, as Chaucer hath it,

"Entuned in the nose full swetely."

We were told by the preacher, that the parish contained ten thousand souls; that there were only ten ministers to watch over these souls, one to every thousand: this was too great a task for one man, and aid was asked, in order that additional ministers might be obtained. Every class in the congregation was appealed to, and labourers were urged to subscribe their coppers. We were rather puzzled to know whether this was meant in good faith or not, and we began to wonder what the building, the massive communion service, the beautiful decorations, the expensive choir, and the superabundance of ministerial assistants had been organized for; and we could only escape improper thoughts by supposing that the value of the ten thousand souls had only been accidentally discovered after the building had been built, the ministers culled, and the choir grown; when, of course, the funds were exhausted.

The moral therapeutics of the metropolis, and of the kingdom generally, do not want the means of application, nor even system, but vitality. They live and they grow, but not commensurately with our wants; and a conviction of this is fastening rapidly and deeply upon the moralists of the metropolis, as witness the energy that is being infused into and the development that is occurring in the services of many religious societies: for example, the establishment of Sunday evening services at St. Paul's and at St. James's Hall, in addition to those at Exeter Hall and at the Abbey; and the institution of simple services in hired rooms, in the hearts of several of the most debased districts of the metropolis (St. George's in the East, Clare Market, &c.), by the clergy of the districts.

How greatly the moral agencies of the metropolis fall short of what is required, may be surmised from the following illustrations, quoted from the Report of the Select Committee of the House of Lords on the Deficiency of Means of Spiritual Instruction and Places of Divine Worship in the Metropolis and elsewhere in the Kingdom (June, 1858):—

"Looking at the actual provision made in London, considered in the large and popular sense as *the metropolis*, it appears that the population being 2,362,236, and the sittings actually provided by all denominations being only 713,561, or 29·7 per cent, no fewer than 669,514, or not much less than half the whole number, are required to raise the sittings to 58 per cent of the population. It appears further that Middlesex, the county which may be considered the central seat of the civilisation, the enterprise, the wealth and power, as well as of the government of this great empire, is actually the very lowest of all the counties of England in the provision made for divine worship by all denominations."—(p. iii.)

Education and police take naturally a primary position in all statistical estimations of moral agencies, as their effects may, with greater certainty than those of other agencies, be determined numerically; but while such evidence clearly shows that the prevalence of education is inconsistent with that of the graver forms of immorality, it also shows that those moral results do not arise from education which might have been hoped for. The same evidence, however, indicates that the cause of this is to be attributed to the imperfect and unsatisfactory character of much of the education prevailing in the country.

From Mr. Fletcher's figures we learn that, at the period to which they refer, the influence of education upon the criminal calendars was very markedly indicated by a general decrease in the number of commitments of individuals who could "*read and write well;*" but, at the same time, there was an increase in the numbers of those of an inferior grade of instruction. Moreover, the decline of absolute inability to read and write proceeded at double the rate among those brought to justice than among those who came to be married. The numbers of criminals of an inferior grade of instruction would necessarily increase with the extension of instruction; but the greater rate of decline of absolute ignorance among criminals than among those who marry is most decisive evidence against the influences associated with much of the instruction prevalent—against the *quality* of the instruction. Of the almost worthless character, whether as to the tuition of the mental or the moral faculties, of the education given in many of the day-schools for the operative classes, abundant illustrations may be found in the Reports of the Government Inspectors of Schools printed from time to time in the Minutes of the Council of Education.

Education (in the ordinary sense of the term), *per se*, is only indirectly a moral agent, and much needless disappointment and doubt as to the effect of education upon the social character of a community has arisen from considering it as directly capable of maturing the moral faculties. This is a grave error. The training of the mental has no necessary relation to that of the moral faculties, and for the right development of the one as the other a specific tutoring is requisite. The great vice of the early training of the present day is, that amongst all classes of schools, high and low, the development of the moral powers is made subsidiary to that of the mental; that neither time nor trouble is spared in cultivating the latter, while the former are too commonly left to grow at hap-hazard, the character and direction of their growth being left to be framed by the example of those around; or once or twice a week, in addition to a scrap of prayer read in the school-room in the morning, a few dogmatical precepts are thrown into the mind and left to take their chance, the master taking no heed as to what kind of ground the seed may have been thrown on. Indeed, in our schools generally, we have no careful, thorough, and practical tuition of the moral as we have of the mental faculties. Need we wonder that many laugh at the moral influences of education altogether?

The beneficial effects of an efficient police force, such as now exists in the metropolis, needs little comment other than what we have already made. The salutary awe entertained of the police-constable by the vicious is a matter of familiar experience:—

"'It ain't no go, as it used to be,' said a housebreaker to me. 'How is that?' said I. He replied (I omit some vulgarities), 'Why, if you get inside a house quietly, don't you see, jist as yer a coming out, there's some policeman a waitin' to ketch you in his arms; and they put such *lots* on at nights, so thick, it ain't no use a trying.' This young man attended my meetings, and appeared to have given up his habits of depredation. He told me lately, 'Mr. Wandecum,' said he, (few pronounce my name correctly,) 'you may believe me or believe me not, but I see things werry differently to what I used to do. I'd rather live upon a penn'orth of bread a-day got honestly, than have lots of *grub* the other way—that I would: not but what there's a deal to be made, perticularly by handkerchiefs, but you're always in fear, yer conscience won't let yer rest; every sound you hears, maybe on the passage or on the stairs, when you're abed, anyhow, you starts up and thinks it's some *peeler* (*i.e.* policeman) come to take yer! It's a miserable life, that it is; there ain't no luck in it. Please the Almighty, I've done with sich ways altogether, and mean to get my bread honestly.' . . . 'Lots of us turns honest now,' said a pickpocket, ''cause it's no go.'"—(*Vanderkiste's Notes and Narratives of a Six*

*Years' Mission, principally among the Dens of London,* p. 12 and p. 23.)

Punishment was formerly the panacea of English law; but experience and increasing knowledge of the nature of vice have taught our judges and magistrates that punishment alone is not sufficient for the repression of vice. Several years ago, Lord Brougham wrote and spoke thus:—

"In reasoning upon the tendency of punishment, and the motive to offend, we have always committed one serious error. We have considered crimes as insulated, and we have regarded each offence as originating in an occasional gust of passion, or view of interest; we have argued as if all criminals were alike in their nature, and all spectacles of punishment, or exhortations to departure from wrong-doing, were addressed to the same minds. Now, nothing can be more certain than that the great majority of all offences committed in every civilised community are the result of immoral character, of gross ignorance, of bad habits; and that the graver sort are committed after a series of faults less aggravated in their character. It follows as a necessary consequence from this proposition, that when the example of penal infliction is addressed to the offender, its deterring effect is very much lessened, because it is addressed to a mind which evil habits have entirely perverted; and thus the guilty-disposed person is to be not merely deterred from doing one wrong act by the fear of punishment, but to be reclaimed from a course of thinking, feeling, and acting into which he had fallen. . . . The effect of punishment in deterring by example is exceedingly feeble upon the whole, and prodigiously overrated in all systems of criminal jurisprudence, as well by philosophers who speculate upon the construction of codes, as by lawgivers who trust to statutes for a protection against offences."—(*On the Influence of Simply Penal Legislation*—Works, vol. viii.)

At the present time, consistently with the views which have been long advocated by Lord Brougham and others, and which have gradually grown up in society, the Bench acknowledges that the law has another and a higher office than the punishment of the criminal, to wit, his reformation; and hence the majority of our great prisons have become moral schools, in which, in addition to punishment, both physical and mental agencies are put into play, by which the criminal may be more surely reclaimed. Now, also, it is becoming well understood and practised that the child who has been bred in criminal habits must not be punished, but *taught.*

We are too apt to forget that our prisons deal mainly with matured and not nascent crime. If we turn to Col. Jebb's last Report on the Discipline of Convict Prisons (1858, p. 106), we find he estimates that from 70 to 75 per cent. of the discharged convicts do not relapse into crime. From this it follows (as we endeavoured also to show from other sources, in the paper already referred to "On the Moral Pathology of

London"), that the majority of the convicts in our prisons are new cases. The lesson is obvious, that the substratum of vice from which crime springs—nascent crime, indeed,—can only be dealt with successfully outside the prison walls.

Religion, "the life-essence of society," as Carlyle hath it, is the chiefest agent contained in the *materia medendi* of the soul; for while directly and immediately appealing to that intuitive consciousness of the Infinite which each individual possesses, and directing that consciousness to the true knowledge of the Godhead, at the same time it rouses into activity the power of moral truth that lies in us, and wings those noble aspirations which stretch beyond time and death. It is the mighty power—a power powerful as appealing from the divine light without us to the divine light within us—which the simple truths of Christianity possess in rousing directly and immediately into fuller play the depths of our religious and moral consciousness, which constitutes the great, the divine force of the Christian religion; but in proportion as these truths are overlaid, hidden, or frittered away by formalism—in proportion as religion is lost in theology—in proportion as the art is merged in the science—in proportion will living Christianity decline. Theology, the logical construction of religious belief, is necessary to the progress and to the perpetuation of true religion; but if theology be suffered to outgrow or to be substituted for the simple primary truths of Christianity —if the product of our own reason be elevated above the precepts of divine teaching, both the practice and the teaching of religion must become sapless, and we shall have a shadow where we thought to find a substance. We write thus, not because we underrate theology, but because we believe the simple primary truths of the Christian religion, common to every Christian sect, to be of higher practical value than any sectional dogmas, however logical they may be.

Consider for a moment the psychical condition of a large number of the metropolitan population. Their minds scarcely admit any other thoughts than those which relate to their daily bread, and their reasons are rarely developed beyond the point requisite to compass their livelihood.

Of what signification to them is a brilliant service (choral responses, theological disquisition, anthem, and so forth)? It is either regarded as a bitter mockery, or a somewhat tiresome scenic show, as a very brief acquaintance with the class in question is sufficient to make evident. People of this stamp can only be roused to religious thought by the direct preaching of the simplest truths of Christianity, aided by those benevolent exertions which make the chief boast of Christianity. Nay, more, to effect good among the lower classes of the metropolis, it is as requisite to understand their habits of thought and forms of expression, as it is for the foreign missionary to study the language and manners of the people among whom he may be cast; and it must not be forgotten that by the lower class of the metropolitan population Christianity is always judged by its doings. A costermonger, addressing Mr. Mayhew, said:—

"I think the city missionaries have done good. But I am satisfied that if the costers had to profess themselves of some religion to-morrow, they would all become Roman Catholics, every one of them. This is the reason:—London costers live very often in the same courts and streets as the poor Irish; and if the Irish are sick, be sure there comes to them the Priest, the Sisters of Charity—they *are* good women—and some other ladies. Many a man that's not a Catholic has rotted and died without any good person near him."—(*London Labour and London Poor*, vol. i. p. 21.)

Again, it may be asked, how great a portion of the vice which exists among all ranks of the educated portion of the population arises from their being taught a mere form of religious belief, the feelings and emotions which vivify that belief being suffered to lie dormant?

The spirit which animates our city missionaries wants infusing more largely among ministers of all denominations. It is not sufficient that the truths of Christianity be uttered from the pulpit. Here is an indication, from the Twenty-second Annual Report of the London City Mission (1857), of what one man may do, actuated by a spirit like that which impelled St. Francis:—

"If the Committee were disposed to refer to missionaries who have been extraordinarily blessed with particular classes of the community, they might perhaps give as an illustration a missionary often called by the name of the Thieves' Missionary, from the usefulness which has attended his efforts with that class, and to whom they are more disposed to allude, as no reference has been made by them to his work for several years past, although it has been remarkable. Since his attention, of late years, as a missionary of the society, has been directed especially to the criminal class, up to the end of March last, he has received visits from 2,625 ruined young men, and from 1,876 ruined young women; and these have paid him in all 84,493 visits. Of these outcasts, 118 have been restored by his instrumentality to their own homes; 153 have been sent to asylums; for 250 employment has been obtained; 74 have been sent abroad to commence a new life in a new sphere; and 40 have been received by Christian ministers as communicants: 576 persons improperly living together have, moreover, by his efforts alone, been married, to each couple a Bible having been presented on marriage by the liberality of Lord Shaftesbury. And in evidence that this great work has not slackened in its importance or results, it may be stated that, during the last year alone, 253 outcast young men and 205 outcast young women were visitors at his abode, who paid to him 9,386 visits. Of these outcasts, 26 were persuaded to marry; 14 were restored by him to their homes; 24 were sent to asylums and reformatories; for 50 work was found; 7 became communicants; and 4 were induced to give up crime, and enter workhouses; while of 5 who died, he was enabled to entertain pleasing hopes. Of the large number of outcast visitors to his house first referred to, 2,319 had been in prison, and the number of their imprisonments had been 9,840; while of those who had not been in prison, more than three-fourths were criminals, and the rest were reduced to a state of vagrancy and filth: 652 of them were under fifteen years of age, and about 4 out of every five of those thus young were brought to the missionary by their parents, which afforded a valuable opportunity, which was not lost, of giving Christian counsel to the parents as well as to the children. In this very bad and low district, there is but one woman now known to be fallen. Very different was its condition in this respect on his appointment to it."—(p. 26.)

As a fuller illustration of the beneficial influence exercised upon the outcast by the one energetic individual referred to in the preceding paragraph, we quote the following from the Twenty-third Annual Report of the Mission:—

"Very soon after one of the missionaries was placed in a district in Whitechapel, he was brought into contact with a considerable number of fallen women. He tried to rescue them from their lost and ruined condition by getting them into the various institutions formed for that purpose in the metropolis, and succeeded in many instances. But he soon found that there was some preliminary place wanted, where hopeful cases might be placed until an opening could be obtained for them into an asylum, without sending them back to their former companions or to a lodging-house, where the good impressions made were so liable to be erased. This was the more necessary, as the Committees of the asylums met but once a week, or, as with the Magdalen, once a month, and even then admission was often impracticable to obtain. In the spring of 1855 it was determined to commence in connexion with the Rescue Society. A small house of four rooms was taken, at which lived a motherly woman, the missionary's first convert. In a year and a half's time, a larger house became necessary, to which the Institution was removed, and where it still is. The part the missionary took in this was—(1), in pointing out the necessity of such an institution being formed; (2), in bringing his local superintendent, the Rev. Mr. Champneys, into contact with the Rescue Society; (3), in assisting in the formation of the Home; (4), in since advising with the matron on every case admitted, she acting under his direction in all she has done in this respect; and (5), in visiting regularly the inmates, imparting religious instruction to them, and counselling with them as to their future course. The matter has required much time and also much experience of the class, neither of which he could have had but as a city missionary. During the three years that this 'Whitechapel Probationary Refuge,' as it is named, has been opened, there have been received into it as many as 257 women, of whom 65 have been sent to asylums, 87 have been placed in service, 60 have been restored to their friends, and but 22 have left the Home of their own accord, leaving 20 there still, and 3 at present in hospitals. How most truly encouraging are these figures, when attentively considered, as results, by the Divine blessing, from the efforts of a single missionary, in so short a period of time!"

In the example furnished by this missionary, we see the best hope of making a salutary impression upon the mass of prostitution existing in the metropolis, and much might be effected by the co-operation of married *women* of mature age in the work. Those who best know the prostitute will acknowledge that her feelings are most readily accessible by an earnest, thoughtful, kind-hearted woman; and both Parent-Duchatelet (*De la Prostitution dans la Ville de Paris*) and Forgier (*Des Classes Dangereuses*) testify to the great influence exercised upon the prostitutes of Paris by the Sisters of Charity. Parent-Duchatelet writes:—

"In order to govern prostitutes, to instruct them morally, and to inspire them with certain sentiments of modesty and good conduct, it is necessary to have recourse to women who either are or have been married. The appellation of married woman, and particularly that of mother of a family, inspires these girls with a peculiar respect, and induces them to submit without murmur to all that may be exacted from them."—(*Op. cit.*, ed. 1st, p. 213.)

We have been recently told of a lady in one of the large provincial towns who has devoted herself to the task of reclaiming the fallen. After many months of unwearied assiduity and never-failing temper, she has overcome the main difficulties to be met with in attempts to gain admission to the residences of these unfortunate women. Taunts, the vilest language, and even slight violence, were alike unheeded. She often got access to the sick, and then her motherly care and attention at last had its effects; and it would be difficult to estimate the good which she is now doing, and the influence she has established among some of the least restrained of the class.*

But, to return once more to the city missionaries, we cannot

* In the article on the Moral Pathology of London (see *Ib.*, No. XII., New Series) we were, by a misprint in the "Judicial Statistics," led into an error concerning the number of prostitutes and known thieves proceeded against in the metropolis in 1857. The number of prostitutes was 9,020, and not 5,911; the number of known thieves was 4,468, and not 10,647. The number of prostitutes known to the police in 1857 was 8,600; and the difference in the number proceeded against from that of known prostitutes arises from the fact that many of these women are often under arrest several times in the year.

refrain from quoting two other illustrations, from the Mission's Twenty-third Report, of the mode in which they do their duties:—

"Another subject, somewhat allied to this (prostitution), has been much of late before the public in the daily journals, and that is the night houses of the metropolis, by which is meant coffee-shops which are kept open throughout the night, professedly for the accommodation of persons requiring refreshment and shelter at such a time. To these, also, the attention of the Mission had been previously directed, and a missionary was specially appointed for their visitation nearly a year since. He leaves his abode about midnight, and continues at his work till the ordinary hours of rising. It scarcely need be said that these houses are the resort almost exclusively of fallen women, and of dissipated and gay, or of destitute and homeless men of irregular habits. In 10½ months, during which the missionary has been at work, he has spent 796 hours in missionary efforts within the walls of these houses, and has distributed there 7,145 religious tracts, chiefly in envelopes. Paragraphs of the Divine Word have been read or repeated there 2,336 times. It is supposed that there are about 250 such houses. Of these 152 are regularly visited by him, and 635 visits have been paid to them. A larger number could easily have been visited by him, but he has already his hands quite full. . . .

"The public-house missionaries, again, have a difficult class of labour, from the character and habits of those visited, and the places in which their work is carried on. Yet one of these, newly appointed this year for the parish of St. Pancras, reports, that in that one parish of the metropolis, 11 miles in circumference, and with 190,000 inhabitants, he has been enabled to visit consecutively every one of the 520 public-houses and coffee-shops therein, without one single exception. And the missionary to the public-houses in the almost equally large parish of Marylebone, who has been longer at his work than the other, reports that this year 18,542 persons have heard the Gospel from his lips within the walls of the public-houses; 1,189 hours have been spent by him this year in such houses; 54 Bibles have been distributed by these, and 21,560 religious tracts. Portions of Scripture have been read or repeated by heart there, on 1,349 occasions; and from such places, 30 adults have been induced to attend public worship, and 45 children to attend schools; 4 fallen females have also been placed in asylums, 2 restored to their homes, and 1 otherwise rescued. One of the fallen ones benefited had attempted self-destruction, and some others were spirit-broken with remorse and care. Of 9 destitute and criminal girls placed by him in reformatories, one was only 13 years of age, but had been apprehended three times for theft, and had been tried at the Old Bailey for picking a lady's pocket. She is now doing well; and this missionary, looking back on his past career of some years' duration as a missionary, adds, 'It has now been my happiness to benefit *several hundreds* of the fallen class.'"

Let us add another extract from the Report:—

"For the preservation of order and property, London has its 7,000 police, at an expense last year (1857) of 444,212*l*. But as yet its religious police of city missionaries are but 350, and its income 32,230*l.*"

We dare not do more than make a brief allusion to the tempting subject of the Ragged School Union, with its 377 Day and Sunday schools, and 44,540 scholars, according to the last Report (1858). That admirable offshoot of the schools, the Shoe-black Brigade, is now a familiar feature of our streets. Two examples from the Report will show in what manner the schools are working.

One school, since its establishment, has saved twenty-one lads from crime, and started them fairly in life. One of these lads, at sea, thus writes to the schoolmaster :—

"I hope you will tell all the boys I advise them to go to sea, for it will make men of them, for I know it has done so with me. I found the Bible and the books you gave me very useful at sea; I am very thankful to you for them, and for all the trouble and pains you took with me when I was at the school. I will, if possible, come to see you, as I should like so very much to see the school again.

"H—— G——."

The following incident is told of the Marylebone-court School for boys under ten years of age, and for girls and infants :—

"One afternoon during the autumn the schoolmistress was taken so seriously and suddenly ill that she was obliged to be at once removed, whilst the school and its contents were left to the 'tender and unrestrained mercies' of the children and neighbours. Not only did the schoolmistress meet with the greatest and kindest sympathy from the neighbours gathered around her by the alarm given of her illness, but on her return to the school, after an absence of some days, she found to her extreme gratification a most unexpected testimony to the moral influence she had exercised, in the fact that not a single article had been touched during her absence, although books, work, slates, and even *money* (left in an open drawer) had been within their power. Less than two years ago, it is confidently felt that this anecdote could not have been given."

We are, perhaps, too apt to believe that our moral therapeutics are mostly applicable to and required among the lower classes of the metropolitan population. The different classes of a community, like that which exists in the metropolis, interlace at so many points, and are so dependent the one upon the other, that no advancement or retrogression can take place in any one class but its effects will be made manifest in the other classes. Thus it happens that every movement made for the benefit of a class is dependent for success in no small degree upon the attitude assumed towards it by the various sections of those classes which are more immediately related to that intended to be benefited. Now, it may be laid down as an axiom that the moral improvement of the lower class is governed by that which takes place in the middle and higher classes; but the moral responsibility of the latter in respect to the former consequent upon this relationship, is yet far from being sufficiently appreciated.

We know that ruinous and filthy houses, in which every room is a separate dwelling-house, breed and propagate vice; that certain courts and alleys are notorious as the haunts of villany and immorality. Has the landlord no responsibility in this matter? Mr. Mayhew tells us of one man who owns nearly a dozen brothels, and who is a member of a strict Baptist Church and the son of a deceased minister!—(*London Labour and London Poor*, vol. i. p. 31.)

Mr. Godwin describes the houses in a court in Clerkenwell as being so dilapidated that few would suppose that they were inhabited, yet the rooms were let at exorbitant rents, and in one of the houses two of the rooms accommodated at night twenty-five persons:—

"The houses in this court belong to a gentleman at Notting-hill, by whom they are let to a chimney-sweeper, who lives on the spot, and then sublets them."—(*London Shadows: a Glance at the "Homes" of the Thousands*, 1854, p. 12.)

Mr. Hill, the Recorder of Birmingham, writes:—

"I look forward with great confidence to the time when an owner who knowingly, or by gross negligence, permits his houses to be occupied for the purpose of carrying on any traffic obnoxious to the laws of his country, will be held himself responsible, and, by fines and other visitations, taught, through his own selfish interests, a due regard to the interests of the public."—(*Suggestions for the Repression of Crime*, 1857, p. 68.)

We know that a main source of crime, pauperism, and vice is intemperance. Have the educated classes no responsibility in this matter—so far, at least, as example is concerned? Are spirit-dealers and the brewers wholly innocent in the matter? Have the electors and their representatives done their duty? Here is a comparatively recent example of one of the inexplicable eccentricities of our licensing system. A young man was brought before the Lord Mayor, in August last, charged with committing a robbery. In the course of the examination the following conversation took place:—

"The Lord Mayor.—Was there nobody at the public-house at the time who can identify the prisoner?

"Watkins (660).—My lord, I went there, but they would not know anything about the matter. The house is a very bad one, frequented by none but thieves and card and skittle sharpers.

"The Lord Mayor.—I thought the licence had been taken away.

"Watkins.—*So it has, my lord. It is now only licensed as a beer-shop, and it is worse than it was when licensed as a public-house.*"—(*Morning Post*, August 27.)

In the intimate relationship which exists between the master and servant, the employer and workman, is that relationship bounded merely by £ *s. d.* on the one side, and service engaged for on the other? Is no higher responsibility involved in the relation? Is it not a reproach on the wealthiest commercial kingdom in the world that so few Akroyds and Prices exist among its employers of labour?

How few of the wealthier classes reflect (to bring charity to its most commercial bearing) with how little cost and trouble to themselves they might be the means of diffusing incalculable benefits among those less fortunate in pelf who surround them! Witness the example set by the Honourable Societies of the Temple in throwing open their gardens to the public during the evenings throughout the finer portions of the year. So remarkable is the testimony of the Temple Gardener, contained in two letters to the *Times*, to the effect exercised upon the children of the lower classes by the privilege of admission to the gardens, that we shall quote them in full, in the hopes that the lesson may not be altogether lost in the approaching summer:—

*To the Editor of The Times.*

Sir,—I read in the columns of *The Times* last week several letters soliciting the favour of the authorities of Gray's-inn and Lincoln's-inn fields being open for an hour or two in the hot summer evenings to the working classes' children in the crowded courts and alleys of the surrounding neighbourhood (I wish they would) for the benefit of the poor, and also to ease us a little of the overwhelming numbers that nightly visit the Temple-gardens. We have too many to be comfortable, and keep the grass alive, this hot, dry season. Still, I don't wish for one moment to interfere with what does not concern me. I only wish to record my practical experience of the class I would grant the boon to, and also show the injury done; and this is founded on upwards of twenty-six years' observation.

This evening I counted no less than 7,000 pass through the gates from six till a little after eight; and when the garden was cleared at twenty minutes past eight, not a flower or even a leaf that I could find was injured. Of course we have several persons to walk round and keep order; and I must here beg leave to acknowledge the usefulness and efficiency of the City Police. I was rather grieved to read the letter of a correspondent in Lincoln's-inn-fields making use of such strong terms towards the working classes, as I find they don't deserve it; for this reason—I find the poorer the children are, the better they behave in the Temple-gardens. The well-clothed, well-fed, and educated youths I am often obliged to keep out, as they only come here for what they term a lark with the girls. They jump on their backs; the girls then take off their caps, and away they run after them to kiss, knock down the little children or any one else in the way; and if spoken to they give a fair share of abuse, and say they will report you for insolence.

Now, I tell these youths to go to the parks and practise their games. These are my greatest trouble to manage here.

Now for the tagrag and bobtail; these I manage remarkably well. They present themselves at the gate:—"Please, sir, can I go in? I have cleaned my shoes." He holds up his leg to prove his case. Another stretches forth his hand—"May I go in? I have washed my hands." Another—"Sir, let me in, please; I have washed my face." And with an innocent, wistful look he throws back or takes off his cap to show he is washed clean. The girls practise the same thing, by saying their frocks and stockings are washed and mended on purpose to get passed into the Temple-gardens. Now, see the good this does the poor children. They get well washed once a day, whereas, perhaps, they would not for a week. They walk round the gardens, and admire the beauties of Nature. It must improve their domestic habits, and also their minds, in seeing flowers instead of filth and every other nuisance that is brought under their notice in the public streets. These are the best behaved in the garden; and I prefer admitting them to the high-spirited, well-clothed, and well-fed young persons, for the above reason: to them it is a grand treat, and they are afraid to do wrong, in case they do not get admitted again. Any person doubting what I have stated, let them come to the garden-gate any fine evening, and judge for themselves. I wish they would turn Old Smithfield Market into a playground; it would greatly relieve these gardens.—Your obedient servant,

Sunday Night, July 12, 1858. SAMUEL BROOME.

---

*To The Editor of The Times.*

Sir,—We have now closed these gardens for the season from the public, to enable me to restore the overtrodden lawn for their reception another year, by sowing fresh seed to make a fresh turf for next spring, which operation I find little difficulty in doing with proper management; but the principal object I have in asking your permission to insert this letter is this,—to show the good behaviour of the working classes, and the benefits resulting from throwing open to them in the hot summer evenings places of this description. I have made a very moderate calculation of the numbers that have taken advantage of this boon, the majority being young children, averaging from two to ten years of age,—210,000. The only damage done to the flowers out of this number all the season was one stock pulled up by a child that strayed from its mother, although there are standing in the garden walks 200 pots of plants, and not a branch or leaf has been destroyed (although on Sunday evenings I have seen the ground so overcrowded with the public, that the dust and steam could be seen to rise like a mist above their heads). The character generally given to the British public is, that they are so very destructive to private property if allowed to be admitted for innocent recreation or exercise for the benefit of their health. This I beg most respectfully to deny, in these gardens, and feel proud to defend my class against those our accusers. The admittance of them into these gardens gives a good deal of inconvenience to benchers and members of the Inn, as they cannot take a quiet walk in the hot summer evenings if they feel so disposed; but I rarely hear a single murmur or complaint from them. On the contrary, some of them will say, "Gardener, this is a lovely sight to see these poor creatures." "How they seem to enjoy our treat!" "I like to see it." "What a pity there are not more such places for them!" "I hope they don't pluck the flowers." "No, sir, they are very well behaved."

There is another feature connected with these gardens that may be mentioned—that is, I cultivate a great number of chrysanthemums, finding they are the best town flower to grow in smoke. This excites immense interest in the working class; they walk round and watch every operation I perform like a cat does the mouse, ask me questions, and beg cuttings of what I have to spare. Others buy them at nurseries, and those who are favoured with a sunny spot take unremitted attention in growing them. This, they tell me, keeps them for hours out of a public-house, from spending their hard earnings, and pouring down their throat that which robs their brain and makes a wretched home. Therefore, I consider the good done by admitting the public unlimited. It is the means of teaching them how to cultivate, as well as of improving their domestic habits, and makes a happy home, creates an innocent rivalry with their neighbours, gives food for the brain, health to the body, and Heaven to the soul to hundreds of the industrious class, for there are no less than nine shows this autumn, and all through seeing the plants in the two Temple-gardens that my kind-hearted masters liberally permit. This working class are happy souls. I could spend all my days among them.

I am your obedient and obliged servant, SAMUEL BROOME.

Temple Gardens, Sept. 8.

Until there be a more sensitive appreciation of the moral responsibilities of the middle and higher classes to the lower class, we shall hope in vain for that marked moral improvement in the latter that we desire. There exists a large amount of ignorance, it is to be feared, among the middle and higher classes of the social and moral conditions of the lower class, and the dispersion of this ignorance must form one of the most important steps in the general moral advancement of the metropolis and the kingdom.

But we may not enter into fuller detail respecting the various moral agencies which exist in the metropolis. We have endeavoured to deal with the spirit rather than with the particulars of its moral therapeutics. Much as the practice of morality may fall short of what it might be, still there is a life in it which, properly nourished, is capable of achieving the highest notions of the moralist and the greatest good of the community. They who have watched the growth, in recent years, of the various efforts which are being made for the moral reformation of the metropolis, cannot fail to have been struck with the gradually increasing energy which is being infused into the different means made use of. This great and cheering sign is not, however, manifested in the metropolis alone, but it is witnessed also throughout the whole kingdom, concerning which the masterly pen of Montalembert has written :—

". . . . I hail again with joy the most significant and most consoling symptom of the actual state of England—I mean the persevering ardour of the flower of the British nation in the pursuit of social and administrative reforms ; of amelioration in the state of the prisons, and that of unhealthy habitations ; in spreading popular, professional, agricultural, and domestic education ; in the augmentation of the resources set apart for public worship ; in the simplification of civil and criminal procedure ; in toiling, in every way, for the moral and material wellbeing of the working classes, not by the humiliating tutelage of uncontrolled power, but by the generous combination of free agency and of every spontaneous sacrifice." — (*Un Debat sur l'Inde au Parlement Anglais.*)

---

